# Applications of Laser Welding in Dentistry: A State-of-the-Art Review

**DOI:** 10.3390/mi9050209

**Published:** 2018-04-28

**Authors:** Asma Perveen, Carlo Molardi, Carlo Fornaini

**Affiliations:** 1 Mechanical Engineering, Nazarbayev University, Astana 010000, Kazakhstan; 2 Electrical & Electronics Engineering, Nazarbayev University, Astana 010000, Kazakhstan; carlo.molardi@nu.edu.kz; 3 Department of Engineering and Architecture, University of Parma, Parma, I-43124, Italy; carlo@fornainident.it

**Keywords:** dental alloys, laser welding, dentistry

## Abstract

The dental industry without lasers is inconceivable right now. This captivating technology has outlasted other possible alternative technologies applied in dentistry in the past due to its precision, accuracy, minimal invasive effect as well as faster operating time. Other alternatives such as soldering, resistance (spot) welding, plasma (torch) welding, and single pulse tungsten inert gas welding have their pros and cons; nevertheless, laser welding remains the most suitable option so far for dental application. This paper attempts to give an insight into the laser principle and types of lasers used for dental purposes, types of dental alloys used by the dentist, and effect of laser parameters on prosthesis/implants. It is apparent from the literature review that laser assisted dental welding will continue to grow and will become an unparalleled technology for dental arena.

## 1. Introduction

Dental alloys are frequently used for either replacing a completely/partially distorted structure or to restoring the disturbed function of orofacial organ. Some of the common applications in dentistry are of decayed tooth reconstruction in particular enamel, dentin with the help of crowns, and fillings, missing teeth replacement by the use of removable dentures, or surgical prostheses and epitheses. This kind of restoration or replacement involves morphological, physiological, as well as psychological re-formation of the patient apart from their main purpose of preventing structural decay and functional failure. The selection of dental alloys needs to consider biological aspects and its technical feasibility. Biological aspects consider chemical and functional biocompatibility other than technical functionality. Dental alloys can be processed inside or outside the mouth of patients [1].

In the dental industry, joining is prerequisite for connecting the same alloys or metal (Titanium to Titanium), the connecting of similar metals to its alloys (AuPt to AuAgCu) or connecting different metal to alloys (AuPt to CoCr). For joining the dental alloys, different natures of thermal joining techniques have been used in the past. Among them, soldering, resistance welding, plasma welding, and single pulse tungsten inert gas welding are pretty common. However, recently, laser welding using an Nd:YAG laser becomes very feasible as the joining technique for the dentist. Whatever process is used to connect these joints, they must satisfy criteria set by standard DIN EN 29333 such as stability up to 350 MPa, free of macro-defects, and being corrosion resistant [2].

One of the most commonly used conventional techniques in dental offices is soldering. Soldering requires general multiple preparation steps for fixing a prosthesis, where each step contributes some sort of distortion. Soldering is prepared using another alloy whose composition is different than the parent material as well as the melting point being much lower than the parent material [3]. Cadmium, gallium, nickel and chromium content of solder may dissolve in the oral environment and cause some health issues for the patient. In fact, the reaction between two different metals and alloys (bimetallism) causes a ‘galvanic effect’, which may be responsible for several oral diseases such as Oral Lichen Planus [4], leukoplakia [5], carious lesions, stomatognathic and/or temporomandibular joint disturbance, periodontal affections and mucous membrane inflammation [6].

Due to weaker joint strength offered by soldering, the prosthesis compromises its mechanical stability when stress corrosion occurs. The soldered part also suffers due to a change of color. Soldering of non-precious alloys experiences a high failure rate when it is in vivo employed [7]. Table 1 gives an overview of different joining techniques used. Soldering proves to be not so convenient for titanium material due to its oxidizing properties although it was pretty successful for precious material like gold. Tungsten inert gas and plasma arc welding are also used for joining dental alloys using an arc with the help of non-consumable electrode, and it can offer high quality joints with better finishing. Nevertheless, these techniques suffer due to heat affected zones profoundly [8]. Moreover, resistance welding that offers joints with no heat affected zone can be useful for joining different materials by using the heat coming from electrical resistance of materials; however, this technique requires overlapping joints. On the other hand, laser welding almost facilitates the welding of all metals including Ti alloys. If welding of Ti is conducted under an argon containing environment, it can enhance the biocompatibility and a corrosion free prosthesis [9]. In addition, laser welding also offers other benefits such as high mechanical strength, reduced distortion due to a narrow heat affected zone, least contamination with oxide free part, faster process time, corrosion resistant joint, and no galvanic effect due to welding without third material. In addition, laser welded fibroblast joints proved to be superior as compared to a soldered one in terms of biocompatibility. It is also reported that laser welding remaining with the joint behaves to be 100% hypoallergenic, non-reactive and insoluble in oral environment [10].

Laser technology appears to be a noncompeting technology as it keeps replacing other conventional surgical procedures in dentistry due to its precision level, accuracy, and productivity. Since its first invention by Theodre Maimann in 1960, lasers have come a long way towards satisfying the technological needs that rises along its way. As per the literature, laser assisted welding in dentistry becomes much more popular at the very beginning of this century, and this paper attempts to provide a comprehensive review of laser welding of dental alloys including underpinning knowledge of laser technology, associated limitations and future trends.

## 2. State-of-the-Art of Laser Technology

Technology of lasers, from the first demonstration given by Maiman [11] in 1960, is almost near to reach a 60-year-old life. This technology, following several steps of improvements, became a fundamental part of the modern life. Lasers have assumed a tremendous impact in a large number of different fields such as: science, engineering, technology and medicine. Their importance extends from basic research to mundane technology. Laser devices can be found everywhere, ranging from consumer devices like CD players, laser printers and tag scanners, to industrial applications like welding, drilling, cutting and texturing. Laser technology, combined with fiber optics technology, holds a major contribution in modern communication systems. Lasers also gained a large amount of importance in scientific applications like microscopy, spectroscopy, interferometry, as well as in medical and biological applications [12]. Combining the capability to be used for machining applications, on a large number of materials, and for soft tissue surgery as well as for phototherapy, lasers have assumed a key role in dentistry [13].

### 2.1. Principle of Laser

**Figure 1:**
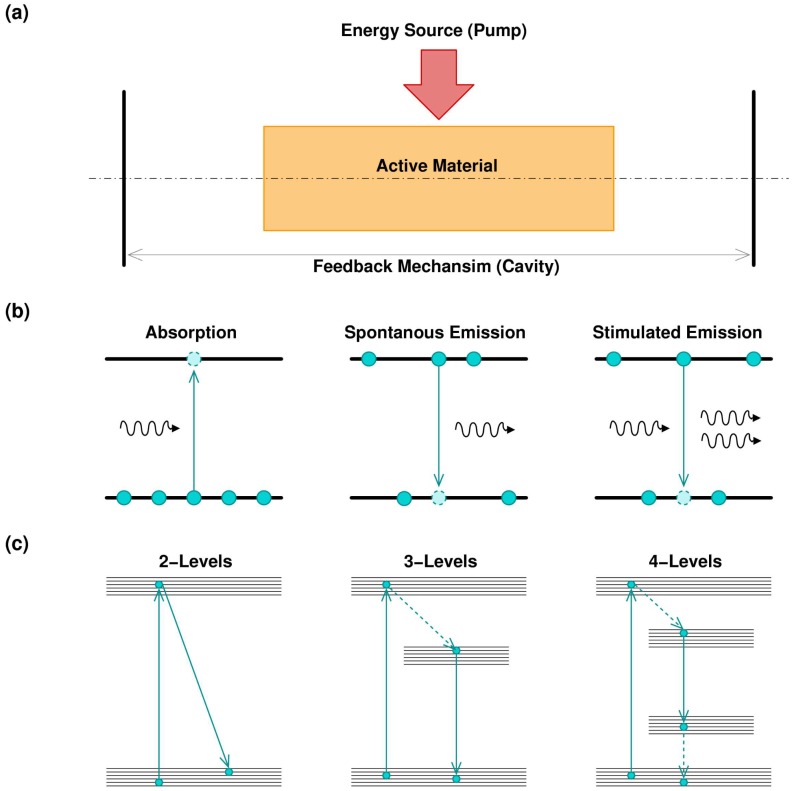
(**a**) Schematic of a laser device, where an active medium is surrounded by a cavity, giving the feedback mechanism, and properly pumped by an energy source; (**b**) Mechanism of light/matter interaction, where the optical amplification is a consequence of the equilibrium among these three effects; (**c**) In real optical media, optical amplification can pass through several energy bands, and some of these passages are non-radiative (dashed line).

The word laser, Light Amplification by Stimulated Emission of Radiation, gives a basic idea of operation principles of laser devices, i.e., the generation of a monochromatic, coherent, collimated and intense beam of light. For extension, the term laser is associated with every device capable of producing a laser radiation. Focusing on laser principles, all laser devices, in order to operate, need to be composed of three fundamental parts: an optical active material, an optical feedback, and an energy supply, as shown Figure 1a [14].

The core of laser devices consists of an optical active material, which is a material capable of absorbing the electromagnetic radiation, represented by photons, at a particular wavelength, thus promoting the atoms of the material to a higher energy state. After that, the material can re-emit photons in a different wavelength. The emission can be spontaneous or stimulated, as shown in Figure 1b. In the second case, the stimulated photon presents the same phase of the incident one, creating a coherent emission. To enhance the mechanism of coherent emission, the active material shall be supported by a feedback system. Optical cavity, where only the wavelengths that are submultiples of the cavity length can create a self-sustained standing wave, represents the typical feedback mechanism. It is worth noting that, in other kinds of lasers, such as random lasers, the cavity is substituted by feedback given by multiple scattering, typical in disordered materials [15,16]. 

The combinations of absorption and stimulated emission, plus the cavity feedback, is therefore the reason of coherent optical amplification. Nevertheless, this is not sufficient. To support laser emission, the active material needs to be constantly maintained in an excited state. For this reason, an external source of energy, usually called pump, is required. Pumps can be of an electrical or optical nature. As an example, in semiconductor lasers, the pump is represented by a current [17], while, in gas lasers, the pump is given by an electrical discharge [18]. Other kinds of lasers use an optical pump, which can be a bright pulsed light or another laser, with worse emission properties. This is the case for solid state or fiber lasers, where a noisy semiconductor laser pumps the system to obtain a higher quality emission beam [19]. The pump absorption/signal emission mechanism can involve more than two energy levels, depending on the material characteristic, as shown in Figure 1c. In this case, some energy decays are not associated with a photon emission, and the energy is transferred to the medium in different ways, such as lattice vibration or thermal stress. Usually, the pump has higher energy with respect to the emitted signal, to exploit intermediate metastable energy levels. This situation can be schematized by modelling materials with three or four energy levels [20].

Laser operation can be characterized by a continuous emission with coherent time on the order of milliseconds or characterized by a pulsed emission with high-energy ultra-short pulses, with pulse length of a few femtoseconds. Pulse emission, obtained by advanced techniques like Q-Switching and Mode Locking [21,22], has the advantage to achieve higher intensity peaks with respect to continuous operation. As an example, a 100 fs long pulse with the energy of 1 mJ per pulse has a peak intensity of 10^15^ W/cm^2^. This high intensity plays a key role to obtain high quality laser machining [23].

It is usual to classify laser devices according to their active material. Thus, lasers can be divided in these main families, each of them with pros and cons:

- ***Semiconductor lasers (diode laser):*** typically obtained using a p-n junction, pumping with an electrical current. Semiconductor laser are versatile and can be found in several wavelengths covering the visible spectrum, the near-infrared (IR) and the mid-IR. The emission power can vary from a few milliwatts to several watts (for industrial application). Pulsed operation can be easily achieved. In general, semiconductor lasers emit beams with poor quality; for this reason, it is common to use this kind of laser as a pump for other kinds of lasers that permits a better output quality.
- ***Gas lasers:*** these lasers are characterized by gaseous gain media. Pumping is obtained by electrical discharges. The emitted beam quality is very high. The main characteristic of gas lasers is to efficiently emit where the other types of lasers have a poor emission. As an example, CO_2_ laser can emit kilowatts of power at 10.6 µm, making this laser suitable for macro-machining operation, welding and cutting [24]. Another interesting example of gas laser is represented by excimer lasers, which can emit hundreds of watts of average power in pulsed operation, in the region of ultraviolet (UV) [25].
- ***Solid-state lasers:*** are lasers where the gain media are crystals or glasses, properly doped with rare earth or transition metal ions. Common media include: Nd:YAG, Nd:glass, Yb:YAG, Yb:glass, Ti:shapphire, Cr:YAG, Er:YAG, and Er:glass. Pumping is achieved by a discharge lamp. The diode pump is also possible to use. With typical emissions in the near-IR region, this kind of laser offers a large span of emission power, with a remarkable beam quality. Pulsed operation, with very short pulses, in case of Ti: sapphire laser, can be obtained.
- ***Fiber lasers***: they are similar, regarding the principle of operation, to solid-state lasers. The doped area is located in the core of the fiber. Thanks to the fiber geometry, this kind of laser offers a better thermal dispersion and a unique possibility to operate in transversal single mode operation [26]. This permits achieving several kilowatts of emission maintaining higher beam quality [27]. Typically, Yb-doped and Yb/Eb-doped fiber lasers are used for industrial applications that require high precision machining. In medical applications, Tm-doped fiber lasers permit a better interaction with the soft tissues [28,29].

### 2.2. Laser Matter Interaction

The energy deposition process of a laser beam, operating in continuous or pulsed operation, into the surface of a solid material, involves interaction with electrons, i.e., electrons are excited and de-excited in a short period of time [12]. According to this consideration, laser–matter interaction is concretized with an extremely fast heating and cooling rates. The result is that, in the heating transient, only a small layer of material is affected by the process of heating while the bulk material remains unaffected.

To explain the reason of this fast interaction, it is possible to consider a simple, but common, case where the matter is represented by a solid with metallic characteristics. Lasers give energy to the surface electrons. The photoelectric effect shall be excluded because photon energy of the common laser used for metal processing (CO_2_, Nd:YAG, Yb:glass…) are too low to trigger the electrons extraction. Thus, the absorbed photons energy involves a conversion to heat energy passing through the excitation of conduction band electrons, which quickly release their energy, exciting a quantum of lattice/molecules vibration, called phonons [30]. This process is in general extremely fast even when the intensity of the laser beam is high. In the general case, when the material does not show metallic properties, i.e., dielectric material, the laser–matter interaction can be more difficult because of the energy required to excite electrons from the valence band. In that case, the use of ultra-short pulses, with high peak intensity can be beneficial. The laser intensity penetration in the material follows, with a good approximation, the following law:

(1) $$I\left(z,t\right)=\;I_{0}\left(t\right)\times \left(1-R\right)e^{-\alpha z}$$

where R is the reflectance of the material and α is the material absorption [30]. In general, the absorption coefficient is very high for most of the material, leading to a complete energy deposition in a few nanometers. The optical coupling is given by the reflectance coefficient. A careful choice of the laser can significantly improve the light–matter interaction. As an example, to machine glass material, a Nd:YAG laser will be quite ineffective since the glass is transparent at its working wavelength, 1064 nm. On the other hand, a CO_2_ laser, operating at 10.6 µm, will be more effective.

The interaction between laser and matter involves change of state from solid to liquid or from solid to vapor, this process is usually called ablation. Since the physical dynamics of the interaction between phases involve a process of re-solidification, the energy deposition can be slowed down. The use of ultra-short pulse laser operation can overcome this obstacle since the pulse dynamics is much faster than the material phases dynamics [23,31].

### 2.3. Laser Welding Principle

During laser beam welding, a concentrated coherent beam of monochromatic light is projected on a small spot of the prepared joint area with the help of some optical arrangement. Usually, inert gas is used for shielding the welded area from atmospheric oxidation. Now, this laser beam starts interacting with the workpiece and, based on the surface condition of the workpiece, some part of laser energy will be conducted inside the workpiece as heat and eventually the temperature of the workpiece is raised. If sufficient energy is available, it may cause melting or vaporizing of the material to be joined together. For welding purposes, a longer pulse appears to be more useful as it can heat the material up to the melting point without vaporizing. For low depth welding close to the surface, conductive welding of joints is carried out with the help of a low power intensity beam. Shallow as well as wide weld nuggets are formed by the conductive heat from the surface [32]. In addition, for transition mode, medium power density results in deeper penetration compared to the conduction mode by creating a keyhole. However, with the increased power, heat density available becomes significantly high enough to vaporize the metal of the weld surface, which comes across at the center of the laser beam, thus creating a keyhole shape where later on the molten metal collapses to form a weld nugget. Therefore, as deep as 19 mm of penetration can be possible with this technique. Keyhole or penetration mode can offer weld joints with more than a 5 aspect ratio [33,34,35].

Nd:YAG laser(1064 nm) is the most widely used one that is having a pulse duration range of 0.5–20 ms, operating within 1–5 KHz frequency with a pulse energy of 8–50 J. Neodymium acts as active gain medium, which is usually doped into yttrium aluminum garnet crystal. Once optically pumped with the help of flash lamps, this laser emits a 1064 nm wavelength light and offers peak power by precisely controlling pulse. A typical average power level can vary from 5 W to 150 W, while the peak power can vary, changing the repetition rate, from 2.5 kW to 7 kW. Another choice in laser welding is represented by a CO_2_ laser, which permits several kW of continuous wave operation, emitting at 10.6 µm. It is worth noting that the CO_2_ laser presents some disadvantages with respect to an Nd:YAG laser, particularly related to the emission wavelength, which requires a special fiber to be delivered from the laser to the sample to be weld. High-power diode lasers (HDPL) can also be used in welding [36]. This family of lasers, emitting at 720–880 nm using AlGaAs junction or at 940–990 nm using InGaAs, consists of monolithic linear or bi-dimensional array of single laser diodes. The emitted power, in continuous operation, can reach several kW. These lasers are typically compact and cheap. However, they present disadvantages related to the poor beam quality and the impossibility of operating in pulsed mode. More recently, with the improvement of fiber laser technology, the use of large mode area Yb-doped fiber lasers has been demonstrated for laser welding [37]. Offering a large emission power of kWs, and the possibility to achieve high quality pulsed emission, fiber lasers represent an alternative to Nd:YAG lasers, in particular in penetration mode welding. 

### 2.4. Other Potential Application of Lasers in Dentistry

**Figure 2:**
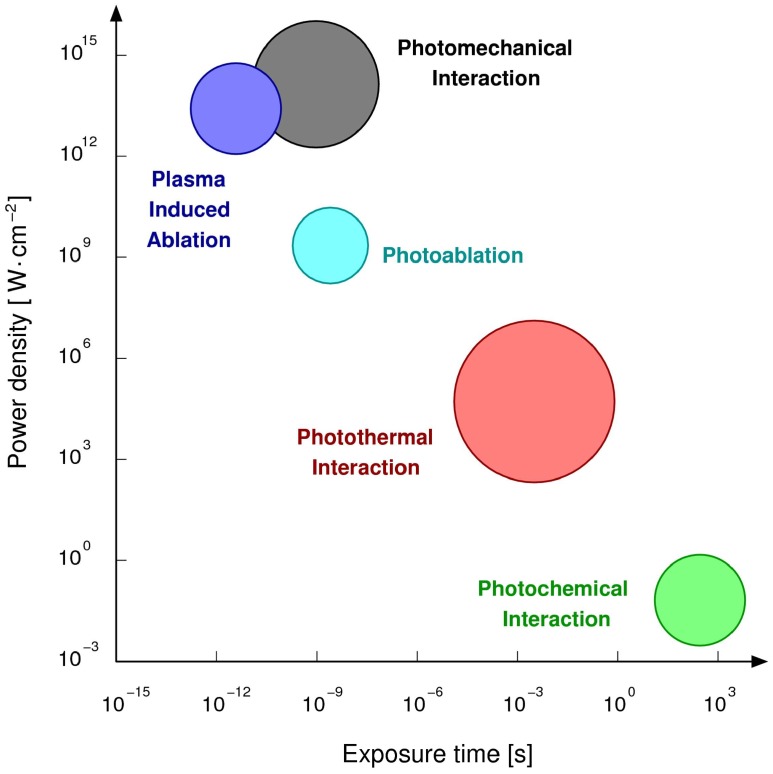
Laser–tissue interaction.

Most of the authors agree that the three main categories of laser-tissue interactions are: photochemical interactions, photo-thermal interactions, photo-mechanical interactions, depending on the time of irradiation and the power density as shown in Figure 2 [38].

In dentistry, the two clinical applications of the photochemical effects are the low level laser therapy (LLLT) and the photodynamic therapy (PDT). The first one, which has been described for the first time by Endre Meister in 1967 [39], allows pain reduction and fast healing process, and it is used after interventions, in orthodontics and in oral medicine [40]; the second one consists of the association of a certain wavelength and a specific chromophore able to absorb the light. It is used in periodontology, endodontics oral medicine and oncology, thanks to its property of selectively killing sick cells, preserving sound tissues [41].

The photo-thermal interactions are the most employed effects in dentistry; in fact, all the dental devices emit with pulse durations of µs and transform the laser radiation into heat, according to the principles previously explained, thus ablating the tissue. The pioneer applications regarding soft tissues surgery successfully exploited CO_2_, Nd:YAG and diodes lasers in order to accomplish important goals such as: bleeding control, pain reduction and disinfection of the operative field [42].

In 1990, Er:YAG was proposed for the treatment of the hard tissues as an alternative to the rotating instruments. This introduced laser technology in conservative dentistry and also in bone surgery. Er:YAG lasers, characterized by spot size smaller than 1 mm, permit a selective ablation of the affected dentin. The surrounding sound tissue is preserved, thus producing an efficient restoration. A large part of literature has demonstrated that in vitro preparation of enamel and dentine by the help of an Er:YAG laser, followed by orthophosphoric acid-etching, increase the effectiveness of the therapy, from the point of view of micro-leakage reduction and bond strength increase [43]. One of the last applications involves the use of laser for the dental bleaching because of its capability to activate the H_2_O_2_ and, consequently, to eliminate the dental stains [44].

At the moment, the use of the photomechanical interactions in dentistry is not yet possible, due to the high costs of the appliances; moreover, some experimental works reached promising results and it is expected that a new chapter of oral surgery, based on the possibility to cut without thermal elevation, will soon start [45].

With the progression of the technology, a dental laser proposed by the market became cheaper and cheaper, smaller and smaller, and more and more performant. Recently, technological innovation has introduced dental diode devices of the same dimensions of a pen and an Ipod, powered by batteries and without necessity of maintenance. Today, the use of lasers in dentistry may be allowed also in patients with pacemakers and the only contraindication regards the utilization existing in pregnant women.

With recent developments in laser technology and reduced expenses, dentists are more motivated to exploit lasers for their clinical practice. With smaller laser pulse and smaller energy, dentists can perform laser applications on their patients with much more safety than before. Minimally invasive nature as well as improved tissue response with better healing has made lasers very attractive technology for the dental industry. Laser technology is shaping the dental industry and providing alternative ways for dentists to perform their treatments. This technology, which is meant for dentistry, will continue to evolve to make itself as user friendly as possible as well as ergonomically plausible.

Restorative dentistry sounds impossible without the aid of lasers: patients reported a high level of satisfaction when treated with Er:YAG lasers [46] and, as demonstrated by the study of Bertrand et al. [47] with the reduction of the pulse durations, the newest advantages will be able to be reached, in terms of quality of the restorations, pain and discomfort reduction, as well as an aesthetic point of view. This technology has a greater relevance when patients are the so-called “Special Needs”, where cooperation is a problem or where general medical problems (coagulation, respiratory or heart failure, kidney transplants, etc.) make it more difficult to give traditional treatments [48,49].

## 3. Types of Dental Alloys and Their Properties

Materials associated with dental industry are categorized into two different classes such as noble metals and base metals. Noble metals, also referred to as precious metals, which appear to be relatively expensive in addition to their chemical composition containing some noble elements. Based on the amount of noble elements’ presence in the metal, dental association has classified dental metals as high noble, noble as well as base metal alloy categories, as shown in Table 2. Gold has a long history to be used in dentistry due to its inert nature, corrosion resistance as well as its durability in the oral environment. However, pure gold has already been replaced by alloys of gold due to its softer nature. Therefore, additions of other metals with gold have been introduced to enhance the mechanical properties.

Base metals refer to those that are not so precious as well as not noble. Common base metals used in dental sectors are titanium, nickel, copper, silver and zinc. However, these base metals have less resistance to corrosion compared to the noble materials, they can increase strength, and be wear resistant when alloyed with noble metals. In general, the base metal alloys should be toxic free or non-allergic to the patient. They should also be corrosion resistant as much as possible and undergo no physical changes under oral conditions. Other physical and mechanical properties include melting temperature, thermal expansion co-efficient, and strength should be satisfactory as per the application. Moreover, fabrication and welding techniques relevant to particular alloys should be available for them to be used in dentistry [50].

### 3.1. Alloys for Crown and Bridge Work

For the fixed crown as well as bridges, gold rich alloys are commonly used. Among these alloys, gold(-silver) (-copper)-PGM(palladium group metal) type offering extra high strength has the applications in removal dentures, long span bridges, clasp attachment and other devices used for fastening. In addition, the gold palladium or the silver palladium systems containing a low amount of gold can also be useful for these above-mentioned applications. These alloys apparently offer 0.1% dimensional accuracy, which is considered to provide pretty good tolerance of a few hundredths of a millimeter [51]. Table 3 provides physical properties of alloys used for crown and bridge restoration. Biocompatibility and corrosion preventing capability of the alloys are the most important properties while choosing the alloys for these applications [52]. However, corrosion resistant properties can be compromised if a non-noble part of alloys is higher in amount, or multiple phases exist, which leads to cytotoxic effects [53,54]. Among these alloys shown in the table, beryllium can adhere oxide on the restoration; nevertheless, it has a carcinographic effect on health other than reducing the corrosion resistance properties [55,56,57]. As per the recommendation from Wataha et al., high noble alloys having the least base metal should be the choice of materials while considering these applications [58].

### 3.2. Alloys for Orthodontics

Alloys used for orthodontics should have a permanent existence in the oral environment where it can maintain biocompatibility for the extended period of time. For restorative and prosthetic dentistry, such as fixed prostheses or crown-bridgework, metal ceramics are widely used. Other than noble alloys, base metals are also used to connect dental porcelain. Most orthodontics appliances use nickel-chromium or cobalt-chromium alloys. However, their behavior in oral environments is thermodynamically unstable, which is correlated with the corrosion resistance capability of the alloy due to the formation of protective oxide film. Ni-Cr alloys are also found to be sensitive to pitting [60]. In addition, orthodontic wires used in the bracket for bonding teeth are made of stainless steel, Co-Cr, beta-Ti and Ni-Ti [61]. Chromium in stainless steel alloys forms a thin transparent layer, which is sufficient enough to provide corrosion protection; however, if a scratch happens on the surface, chloride ions containing an oral environment resists recreating the chromium oxide layer. As a result, stainless steel can be susceptible to pitting corrosion [62].

### 3.3. Alloys for Implants

Alloys for implants should have adequate biocompatibility, toughness, resistance to corrosion, wear and fracture. In addition, they need to satisfy the strength criteria as well to sustain occlusal forces without permanent deformation, and low modulus for optimum force transfer [63,64].

Gold alloys used to be one of the most used materials to be exploited in dental implants due to its extremely good corrosion resistance. One of the important developments in the restorative dentistry happened with the fusion of porcelain veer to metal substrate, which proves to be satisfying while considering both aesthetic as well as technical requirements. Implants made of porcelain veneered Au-Pt alloy has been successfully experimented on without inflammation as well as growth of bone materials. Apart from this, gold alloys also meet the requirements similar to yellow gold ones [51]. Titanium and its alloys are also reported to be functioning at a similar standard as gold [65,66,67]. Moreover, various metals including stainless steel (bone plates and screws), cobalt chromium (cast partial denture frameworks) was also infrequently used [68,69]. However, corrosion resistance of stainless steel as well as cobalt chromium is pretty inferior compared to Ti. Although ceramics have been introduced as coating materials for implants, some of the ceramics materials such as Yb-stabilized tetragonal polycrystalline zirconia (Y-TZP) is found to be suitable as dental implant substrates due to its improved mechanical properties (fracture toughness) [70]. Table 4 and Table 5 give an overview of implant material used in dentistry and different lasers used for them.

## 4. Laser Welding of Dental Alloys

In 1970, in an initial report, Thomas et al. narrated the advantages of laser welding for a dental prosthesis. Their report described the accuracy advantage of laser welding over other traditional processes as well as un-annihilated anatomic interproximal regions due to avoidance of heat distortion and mold transfer [9]. Due to higher costs of gold alloys, dentistry explored other possibilities of using metal alloys. However, it appears that a nonprecious alloy bridge experiences a greater rate of failure in the mouth compared to soldered gold. Therefore, with the invention of different dental alloys, processing of these alloys needs to be developed as well. Apotheker et al. conducted a comparative study on laser welding of nonprecious materials with those joints by soldering and suggested stronger welded joints in the case of Nd-YAG laser welding [7]. Tambasco et al. also demonstrated applicability of laser welding in a fixed partial denture instead of soldering [72]. Goldman et al. [73] mentioned in their reports about the potential usage of lasers (CO_2_ and ND: YAG lasers) in tooth restoration and oral surgery [73].

Bertrand et al. investigated the laser welding (Nd:YAG Laser) of Ni-Cr-Mo and Cr-Co-Mo alloys for having more information about its accuracy, and quality as well as reproducibility. Their findings suggested excellent weldability for Co-Cr alloy; however, a higher amount of carbon and boron causes poor weldability for the Ni-Cr alloy. In addition, both the alloy shows increased hardness on the welded area due to a heat treatment effect of lasers [74]. In addition to this study, Bertrand et al. also conducted another investigation on FeNiCr dental drawn wires to identify the laser parameters for better weld quality. Their findings suggested laser power of 0.8–1 kW for better penetration where welding time can be adjusted for the selected power [75]. Bertrand et al. (2007) also investigated the effect of laser parameters on Ti grade 1 with a pulsed Nd:YAG laser. Parameters considered were pulse shape, pulse frequency, focal spot size and output considered was after ablation microstructure. Their findings suggested that rectangular shape pulse and pulse frequency of 1–2 KZ provide better results. Increased pulse frequency can lead to increased oxygen contamination in the welded region [76].

**Figure 3:**
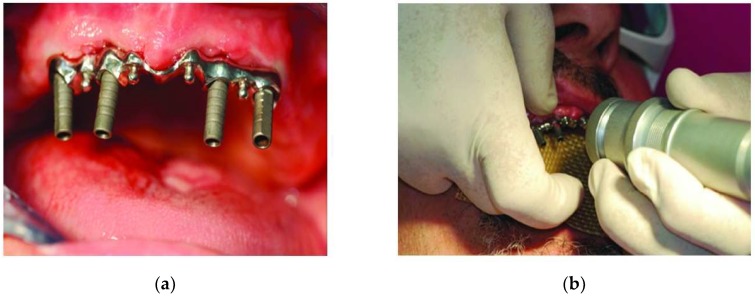
(**a**) Bar inserted in the abutments; (**b**) intraoral laser welding. Reproduced with permission from [77].

Brudvik et al. demonstrated the potential of laser welding in removing partial denture framework. For Co-Cr alloy [78], Fornaini et al. applied the Nd:YAG laser to investigate in vitro efficiency of metal welding for dental prosthesis. In their study, intraoral laser welding (9.9 mJ, 1 Hz, 15 msec, 0.6 mm spot size) was undertaken to join a bar to the abutments screwed to four implants that were already connected to the edentulous maxillary arch of an elderly patient (Figure 3). This process of intraoral welding took 47 sec, and it has opened up new possibilities to apply risk free intraoral welding for dental purposes as well as to reduce the inherent inaccuracies involved with a conventional costly, time-consuming impression taking process [77]. Thus, the application of laser technology not only enhances the dimensional accuracy, but also increases the process efficiency without compromising the strength of weld joints. In another study, Fornaini et al. also compared the results of laser welding generated in laboratories and dental offices using an Nd:YAG laser device and demonstrated insignificant variation in the produced weld [79].

**Figure 4:**
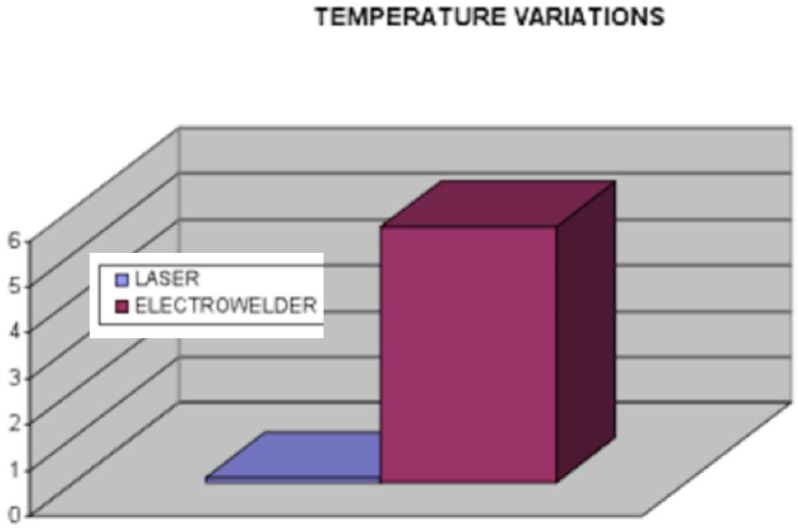
Comparison of the two devices’ temperature variations. Reproduced with permission from [80].

Fornaini et al. also investigated the comparative performance study of laser welding and electric welding on implants of pig jaws. Each welding process was applied to weld Ti abutment attached with implant to the Ti bar on each side of pig jaws without filler materials. Process parameters used for electro-welder were 25 V, 50 Hz, 312 J, whereas, for the Nd:YAG laser, they were the frequency of 1 Hz, energy of 9.85 J, and pulse duration of 15 msec. As per the result (Figure 4), temperature rise during electro-welding is much more significant than laser welding. As per the literature, temperature rise above 47 °C is considered critical for the bone; therefore, laser welding technology offers a promising lifespan for the implant and prosthesis [80]. In addition, electro-welding cannot be used in patients having pacemakers due to the possible electric interference with this device [81]. On another similar investigation, Fornaini et al. also established that joint strength achieved by electro-welding process for Cr-Co-Mo plate is significantly lower than the laser process [82], which is compliant with the observation suggested by Baba et al. [83]. Degidi et al. also conducted a similar investigation on human beings where an intraoral Ti bar was welded and loaded on the same day of surgery. Their findings suggested successful restoration of a permanent prosthesis, which is maintained by a welded Ti framework intraorally [84].

**Figure 5:**
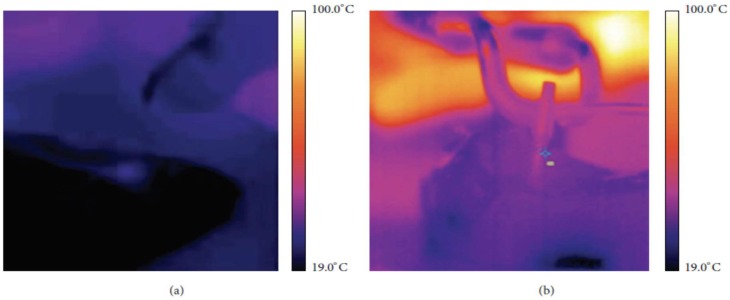
Thermal camera images of laser welding (**a**) and electro-welding (**b**). Reproduced with permission from [85].

Fornaini et al. also conducted a comparative study between laser welding and an electro-welding process to join a Ti implant placed in the pig jaws with a Ti bar. Although experimental observation suggested higher temperature elevation for electro-welding compared to laser ones, the level is still below the critical zone (Figure 5). Therefore, both the process has the potential to be used without any risk of damaging the surrounding biological structure [85]. Fornaini et al. also demonstrated successfully the application of fiber lasers in welding of broken prosthetic and orthodontic therapy inside the dentist office [86]. On another study, Fornaini et al. also investigated soft tissue management during a laser welding operation. In their study, 810 nm and 980 nm laser diodes as well as 1064 nm Nd:YAG solid-state laser were used for maxillary vestibular and lingual frenectomies, for surgical exposure and alignment of ectopic or retained teeth, and for gingival overgrowth re-contouring. The usage of such wavelength laser helps in performing the job without any local anesthesia, which in turn helps to manage the soft tissue inside the orthodontic treatment. Their findings suggested reduced operating and post-operative healing time as well as increased comfort for the patience [87]. Another similar study done by Fornaini et al. agreed with the result provided in earlier studies. However, this later study presented results that exploited, 532, 810, 980, 1064, 2940 and 10600 nm wavelengths with different treatment times. Their results suggested several important considerations such as stronger adhesion of bracket to enamel, and detachment of porcelain brackets without damaging it on top of the reduced needs of anesthetic injection during laser irradiation [88].

Watanabe et al. studied the effect of laser welding on the cast plates of Ti, Ti-6Al-7Nb, Au, and Co-Cr alloy joint, using an Nd:YAG laser under argon gas shielding. Operating parameters utilized during the study were 10 ms pulse duration, spot diameter of 1 mm and 200 V voltage. As per their investigation, the failure load for CP Ti and Ti-6Al-7Nb significantly increased from non-gas to a gas shielding process, whereas Co-Cr alloys exhibited an opposite trend [89].

Iwasaki et al. investigated the dental alloy (Ag-Pd-Au, Au-Pt-Ag) and Ti alloy with the help of butt joints. A Nd:YAG laser, operating in single pulse mode, driven by currents, respectively, of 150, 200, 250, and 300 A, emitting with spot diameters of 0.6, 0.9, 1.2 mm, and pulse duration 10 ms has been used. Their findings suggested increased hardness for welded area than base materials as well as lower fracture toughness for dissimilar butt joints than similar material joints. Reduction in fracture toughness is due to the cracks and pores formed in the weld zones and this is the result of insufficient melting and mixture between components with different properties. Resulting low strength weld joint are considered to be the consequences of cracks, porosity and precipitates generated in the welded region [90]. Nishio et al. also investigated several dental alloys for evaluating the performance of the butt joint. As per their investigation, similar material welding provides adequate strength as well as a better welding condition. A butt joint of cobalt chrome alloy to platinum along with filler materials exhibits positive results; however, a butt joint of cobalt–chrome alloy to gold/silver palladium alloy as well as cobalt–chrome alloy containing titanium to a platinum-added alloy without filler materials offer better results [91].

Goran Sjögren et al. investigated laser welding of pure titanium, which can be exploited for osseo-integrated implants. Compared to a brazed gold alloy, a laser welded Ti part shows ductile fracture and other favorable mechanical properties [92]. On another study, Chai et al. also investigated pure Ti for dental restoration to optimize the laser welding parameter. Using a three-dimensional response curve, optimum parameters (pulse duration, voltage) for response variables such as tensile strength, yield strength, and percentage elongation are evaluated [93]. Watanabe et al. also investigated laser welded gold alloy and recommended to conduct heat treatment on the laser welded cast gold alloy for increasing the hardness and mechanical strength [94]. Einer et al. also conducted comparative investigation on laser welded casts and wrought Ti with brazed gold alloy. As per their findings, laser welded Ti and brazed gold alloy offers similar strength suggesting the application of Ti in dentistry; however, both joining techniques aid in reduced ductility [95]. Walter et al. did a comparative study between titanium and gold-alloy fixed partial dentures (FPDs) for 47 patients and reported no significant difference in the survival distribution [96].

Russel et al. investigated thermal modeling of laser welded Ti part for dental restoration using one-dimensional finite difference analysis. As per their finding, low conductivity of Ti causes a lack of laser penetration depth while damaging the surface extensively, which is not the case for gold. Nevertheless, time-elapsed multiple pulses can eradicate this problem by giving enough time for the excess energy to diffuse into the deeper zone of the materials [98]. Watanabe et al. also investigated a laser effect on the surface preparation. In their study, they prepared gold and titanium samples using air-abrasion with 50 μm Al_2_O_3_, colored with a black marker, ground with SiC particles and mirror polished for laser welding (Nd:YAG laser) operation. Their result suggested that increasing voltage increases the laser penetration for all the samples. The Ti sample containing a black marker as well as air-abrasion demonstrated deeper penetration than other samples; nevertheless, for welded gold joints, penetration seems ineffective by surface preparation [99].

Waddel et al. studied an extensive literature review on a failure mechanism of a soldered bar part used in the removable implant over dentures. Their findings suggested a low failure rate for inter-abutment bars, whereas a higher rate of failure for bars with distal cantilever extension. In addition, they also studied fixed prosthodontics for finding the potential parameters contributing to the failure of solder joints attached to the bar and reported fatigue failure stresses as one of the contributors [100].

**Figure 6:**
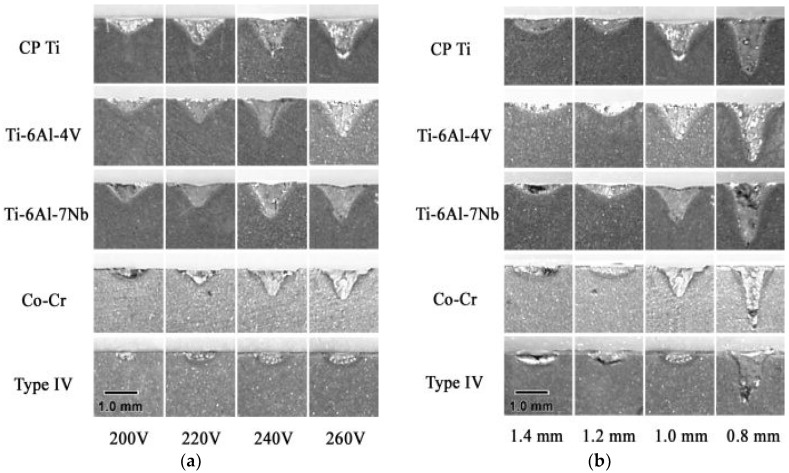
(**a**) Photographs of cross sections at voltage of 200–260 V (14.2, 17.9, 21.1, 29.3 J) and spot diameter of 1.0 mm (Nd:YAG laser, pulse duration 10 ms); (**b**) photographs of cross sections at spot diameter 0.8–1.4 mm and voltage of 260 V(29.3 J)(Nd:YAG laser, pulse duration 10 ms). Reproduced with permission from [97].

Santos et al. investigated laser welded Ag-Pd-Au-Cu alloy dental implant prostheses to find the effect of laser welding. Their observation suggested extreme corrosion resistance of a welded area compared to base metals under simulated mouth cavity environment. In addition, finer grains were observed in the welded area due to high speed cooling [101]. Baba et al. conducted a comparative study on penetration depth variation for pure titanium (CP Ti), Ti-6Al-4V, Ti-6Al-7Nb, cobalt–chromium alloy (Co–Cr) and Type IV gold alloy using a laser welding technique (Figure 6). Laser welding parameters such as 16–340 V, spot diameter of 0.4–1.6 mm, and pulse duration of 10 ms were used to weld the butt joint from each materials. Their experimental findings suggested that, with the increased voltage and decreased spot diameter, penetration depth increased for all the materials [97]. James et al. investigated different prostheses. Specifically, they compared the performance of the traditional prostheses, obtained with a cast framework, with those prepared by laser welding the prosthesis structure with a fixed partial implant. Their findings resulting from three years of observation does not show any significant difference [102]. Liu et al. investigated the Nd:YAG laser output energy effect on Ti joint strength welded by lasers. Their findings verified an insignificant strength difference for a laser welded part joint under different current conditions [103].

White et al. investigated the laser application on intraoral soft tissue surgery, particularly comparing the performance of conventional scalpel with an Nd:YAG laser. Their findings suggested no significant difference on postoperative inflammation, pain and lessening of pocket depth or general reduction of time of treatment. Nevertheless, laser surgery resulting in a lesser amount of operative as well as postoperative bleeding can be done with a lesser amount or no anesthesia [104].

## 5. Laser Welding Phenomena

**Figure 7:**
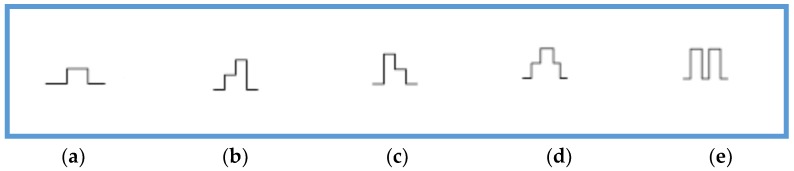
(**a**) rectangular pulse—mainly for pure metal and Ti and results in a smooth surface; (**b**) positive slope pulse—with higher pulse frequency, it offers a good homogenous surface with reduced micro-hardness; (**c**) negative slope pulse—mostly suitable for CoCr alloys and AgPd alloys; (**d**) this pulse shape is suitable for joining hybrid alloys but not well for grade 1 Ti due to high micro-hardness; (**e**) this shape is suitable for welding with high depth and not suitable for Ti [76].

Laser welding technology involves a high initial investment on top of other operating and maintenance costs. Laser welding suffers due to its limited depth capacity. Previously used dental alloys such gold as well as unalloyed titanium can be potentially joined using this technology. Nevertheless, the joining of Ti experiences problems due to its high melting point, low thermal conductivity, its strong affinity towards oxygen, and reactivity [92]. Laser welding with argon shielding can overcome such relevant issues [105,106]. With the invention of Nd:YAG devices, laser welding can be much more tuned by controlling more operational parameters. However, a specific pulse shape of lasers such as rectangular ones during Ti welding appeared to be reducing the generated thermo-mechanical stresses as a result of refined microstructure and thus contributed to the reduced crack generation [76]. Figure 7 shows the various pulse types for Ti and other alloys.

Laser welding of nonprecious materials also experience other issues. Internal defects such as porosity, voids and cracks are pretty much persistent problems associated with laser welding. The reason behind these defects is related to rapid cooling and solidification characteristics of a laser process, which as a result contributes towards a brittle nature of weld. It also causes significance changes in microstructure and physical properties. A laser welded portion offers more brittleness than its parent materials [74,107,108,109].

While investigating a Ti alloy, Bertand et al. reported on the reduced depth of welding and increased micro-hardness for a repetition rate of 10 Hz. The reason behind their findings is the low conductivity of Ti, which does not allow quick heat transfer to the inner side of the plate, thus resulting in the plasma creation on the surface. Therefore, excess energy cannot increase the surface temperature up to the melting point [76]. Ti dental prostheses when welded by lasers offer a limited depth of penetration as well as extensive surface damage. Russel et al. also modeled a thermal effect of laser welding on Ti and gold dental restorations with the help of a one-dimensional finite difference analysis. Their findings reported low conductivity of Ti to be responsible for such problem and gold is free from such limitation. Nevertheless, application of multiple pulses with time elapsed appears to be solving this issue [98]. Bertrand et al. investigated a Pd-Ag-Sn alloy, which suffers from hot cracking and a Co-Cr-Mo alloy, which are susceptible to internal defects such as voids and small porosities. Their study exploits a different shape of laser pulse to annihilate the internal defects using a Nd:YAG laser. Among the possible pulse shapes, their study suggested using a rapid slope pulse shape for Co-Cr-Mo alloys because of the poor reflectivity offered by the material, and a slow rising slope for a Pd-Ag-Sn alloy due to its high laser beam reflectivity, as it allows for a slow cooling ramp, which results in better control over the solidification process [110].

Another issue related to a laser welded part comes from their service in a fluoride containing environment. Fluoride used for dental decay prophylaxis (e.g., toothpastes) causes corrosion of a Ti welded joint as well as plays rough to the protective TiO_2_ formed on the Ti part. These phenomena may influence the cracking and fatigue behavior of Ti while the part is under a fluoride environment. Boere et al. researched Ti and reported on the enhanced corrosion of Ti under the acidic environment in the presence of fluoride [111]. Huang et al. investigated cracking tendency and fatigue performance of Ti while welded by an Nd:YAG laser. As per their research findings, although the increased welding energy contributes in increased elongation and fatigue life with decreased tensile strength, the fluoride environment nevertheless causes increased cracking tendency and reduced fatigue life up to welding energy of 11 J. Nevertheless, welding energy of 15 J caused no reduction to the fatigue life of Ti joints [112]. On another study, Huang et al. also reported on reduced micro hardness, and breaking strength tendency of Ti welded joints due to increased laser voltage [113]. Huang et al. also investigated the corrosion resistance behavior of pure Ti under acidic 1% NaCl solution (pH = 6) environment with respect to variation of fluoride concentration, as well as elastic tensile strain with the application of electrochemical impedance spectroscopy (EIS) measurement. Increased NaF concentration as well as elastic strain are the reason behind increased corrosion rate [114].

Nakagawa et al. also researched corrosion characteristic of titanium, Ti6Al-4V, Ti-6Al-7Nb Ti0.2Pd and alloys under a wide range of pH and fluoride concentrations. Their findings suggested increased corrosion resistance offered by Ti0.2Pd due to its surface enrichment with Pd, thus enhancing re-passivation of Ti [115]. Matono et al. conducted a similar investigation on Ti, Ti-6Al-7Nb and Ti-6Al-4V alloys and Ti-0.5Pt under a Acidulated Phosphate Fluoride (APF) solution (0.05% to 2.0% concentrations). Their findings suggested that, although Ti-0.5Pt appears to be a more corrosion resistant alloy than others in 0.05% APF solution, in 2% APF solution, dissolution of Ti from this alloy surface increased significantly [116]. On another study, Nakagawa et al. also considered the effect of dissolved oxygen apart from fluoride concentration on titanium, Ti-6Al-4V and Ti-6Al-7Nb alloys, Ti-0.2Pd and Ti-0.5Pt alloys. They reported on reduced corrosion resistance of Ti and Ti alloys under reduced oxygen concentration; nevertheless, Ti-0.2Pd and Ti-0.5Pt alloys do not compromise their corrosion resistant ability under low oxygen and fluoride environment [117]. Watanabe et al. studied titanium based orthodontic wires under fluoride prophylactic environment and reported on a surface color change of a titanium–molybdenum wire due to its high Ti content [118]. A similar study was conducted by Pröbster et al. [119]. Zavanelli et al. [120] studied laser repaired pure titanium (CP Ti) and Ti-6Al-4V alloy under different media such as air, synthetic saliva and fluoride synthetic saliva and reported on the reduced fatigue life under wet conditions. In addition, laser welding compromised the life expectancy of the part significantly.

With the introduction in prosthetic dentistry of the so-called “metal-free” materials, other kinds of problems arose for the practitioners; in fact, the risk of fracture, which is not negligible [121], may be reduced by an adhesive cementation and the condition for a good performance of it is to have a ceramic surface that is very rough [122]. Several methods were proposed for this purpose, the most popular being the utilization of hydrofluoric acid [123], but, unfortunately, its employment is not free from disadvantages [124]. For this reason, the utilization of the laser technology seems to be a good way to characterize the ceramic surface demonstrating, at the proper parameters, to preserve it from cracks, melting and fissure formation [125].

## 6. Future Trends

Laser application in dentistry has been there since the end of last century. Nevertheless, its utilization was mainly limited to specialists and researchers due to its high installation costs as well as limited control on the thermal effect of lasers on soft/hard tissues. With recent developments in laser technology and reduced expenses, dentists are more motivated to exploit lasers for their clinical practice. With smaller laser pulse and smaller energy, dentists can perform laser applications on their patients with much more safety than before. Minimally invasive nature as well as improved tissue response with better healing has made lasers very attractive technology for the dental industry. Laser technology is shaping the dental industry and providing alternative ways for dentists to perform their treatments.

As described before, the greatest difficulties and problems related to the usage of laser technology for dental welding consist of the difficulty of arranging the parameters in the function of the material used, its thickness, the gap dimension and the filler alloy. Moreover, up to now, Nd:YAG laser welding devices remain bulky and require a high cost of installation and maintenance. For such reasons, the realization of small and cheap devices that are easy to use with a minimal training phase would be a great opportunity for dentists to weld metals directly in their clinics.

The employment, in the field of laser dentistry, of diode lasers, where the active medium consists of a semiconductor, has created some sort of revolution. By considering their small size and cost, the reduced size of the pumping system, the flexibility offered by the optical fiber delivery system, diode laser technology has been able to reach most of the dentists and is becoming very popular. Even if the common use of diode lasers, emitting around 810 and 940 nm, is for soft tissue applications, due to the great absorption in hemoglobin, different wavelengths have recently been proposed [126]. In this context, a feasible possibility of a diode laser application for welding has emerged in the field of dentistry. In fact, diode lasers emitting at 1064 nm, operating in CW operation or in chopped mode, with a high duty cycle, can be effectively taken into consideration to weld metals in dentistry, thus representing an interesting approach to improve the use of laser welding technology. This technology, which is meant for dentistry, will continue to evolve to make itself as user friendly as possible as well as ergonomically plausible.

## 7. Conclusions

Over the past few decades, several technologies have been employed to solve existing issues in dentistry. Laser technology has thus far been the latest addition among these technologies that clearly has made a remarkable impact and hence replaced some of the conventional techniques due to its high precision level, biocompatibility and minimal side effects. Laser welding is one of the very recent yet versatile techniques used in dentistry, which is capable of manufacturing good quality weld joints with remarkable consistency. It has offered greater advantages such as reasonable hardness, reduced heat affected zone and toughness over other compatible technologies available so far. Most importantly, this technology offers dental patients an intraoral surgery with limited anesthesia or without anesthesia, better comfort level, speedy recovery and aesthetic satisfaction. In light of a huge demand in laser welding technology, this paper has demonstrated the fundamentals of laser technology along with laser welding principles, a brief overview of laser welding for dental materials and a laser welding phenomenon in a systematic manner. Laser welding technologies that currently available are already high-end technology. Further areas of improvement may combine the diagnostic and therapeutic laser welding technologies in one single device. There is also potential for robotic assisted dental laser welding instruments for finer laser welding operation. It will be not surprising if laser welding technology takes the place of most of the conventional alternatives by the middle of the current century.

## Figures and Tables

**Table 1:** Evaluation of different joining methods.

Method	Torch Soldering	Post (Ceramic Furnace) or Infrared Soldering	Spot Welding	Plasma Welding	Laser Welding	Tungsten Inert Gas (TIG)
Equipment expenditure	Small	Moderate	Small	Moderate	High	Moderate
Application depth, versatility	high	High	Small	medium	High	High
Educational prerequisite	Moderate	Moderate	Small	medium	Medium	Medium
Biocompatibility	Small	Small	Good	Moderate	Good	Good
Heat Affected zone	large	large	Small	medium	Very small	Very small

**Table 2:** Definition of metal alloys according to the percentage of noble metals.

Type of Alloy	Noble Metal Content
High noble	Minimum of 40% gold and at least 60% of noble metal elements Example: gold-platinum-palladium, gold-palladium-silver and gold-palladium
Noble	≥25% by weight noble metals. Example: palladium-silver, palladium-copper-gallium, and palladium-gallium
Base metal	≤ 25% noble metal Example: Nickel-chromium and cobalt-chromium
Ti and its alloys	Ti content more than 85%

**Table 3:** Typical physical properties of percaline fused to metal (PFM) alloy. Reproduced with permission from [59].

Alloy Group	Vicker Hardness	Modulus of Elastic (GPa)	0.2% Proof Stress (MPa)	Tensile Strength (MPa)	Specific Gravity (g/cm^3^)
High Gold	200	90	480	580	18.1
Gold-palladium (no silver)	240	124	550	800	14.8
Gold-palladium-silver	200	110	600	680	14.9
Palladium-Copper	275	96	800	851	10.6
Palladium-Silver	260	138	650	810	11.4
Nickel-Chromium	240	160	360	580	8.6
Nickel-Chromium-Beryllium	240	192	552	1138	7.8
Cobalt-Chromium	310	210	480	720	8.5

**Table 4:** Materials used for dental implants. Reproduced with permission from [71].

Implant Material	Common Name or Abbreviation
I. Metals	
Titanium	CpTi
Titanium alloys	Ti-6A1-4V extra low interstitial (ELI)
	Ti-6A1-4V
	Ti-6Al-7Nb
	Ti-5Al-2.5Fe
	Ti-15 Zr-4Nb-2Ta-0.2Pd
	Ti-29Nb-13Ta-4.6Zr
	Roxolid (83–87%Ti-13–17%Zr)
Stainless Steel	SS, 316 LSS
Cobalt Chromium Alloy	Vitallium, Co-Cr-Mo
Gold Alloys	Au Alloys
Tantalum	Ta
II. Ceramics	
Alumina	Al_2_O_3_, polycrystalline alumina or single-crystal sapphire
Hydroxyapatite	HA, Ca_10_(PO4)_10_, (OH)_2_
Beta-Tricalcium phosphate	β-TCP, Ca_3_(PO4)_2_
Carbon	C
	vitreous
	low-temperature isotropic (LTI)
	ultra-low-temperature isotropic (ULTI)
Carbon-Silicon	C-Si
Bioglass	SiO_2_/CaO/Na_2_O/P_2_O_5_
Zirconia	ZrO_2_
Zirconia-toughened alumina	ZTA
III. Polymers	
Polymethylmethacrylate	PMMA
Polytetrafluoroethylene	PTFE
Polyethylene	PE
Polysulfone	PSF
Polyurethane	PU
Polyether ether ketone	PEEK

**Table 5:** Lasers used for dental materials [12].

Materials	Laser Type
Ti and its Alloy	CW–CO_2_, Pulsed Nd:YAG laser, Fiber laser, Yb:YAG ytterbium laser
Ceramics	CW–CO_2_, KrF excimer laser, pulsed YAG laser
Steel and its alloy	Pulsed Nd:YAG, CW-laser, Photolytic iodine laser, CW–CO2 and diode laser
Al alloy	Pulsed Nd:YAG laser, CW–CO_2_, Fiber laser
Gold	Semiconductor laser, Nd:YAG laser
